# Transforaminal Epidural Block and Erector Spinae Plane Block to Manage Acute Zoster-Associated Pain: A Retrospective Case–Control Study

**DOI:** 10.3390/medicina60030453

**Published:** 2024-03-09

**Authors:** Hyojung Soh, Yeona Ko, Jungwon Shin, Eung Don Kim

**Affiliations:** Department of Anesthesiology and Pain Medicine, Daejeon St. Mary’s Hospital, College of Medicine, The Catholic University of Korea, Daejeon 34943, Republic of Korea; thgywjd1106@gmail.com (H.S.);

**Keywords:** herpes zoster, transforaminal epidural, erector spinae plane block, postherpetic neuralgia, zoster-associated pain

## Abstract

*Background and Objectives:* Achieving adequate pain reduction in the acute phase of herpes zoster is essential for preventing postherpetic neuralgia (PHN). For this purpose, appropriate antiviral medications, oral analgesic medications, and various nerve block methods could be applied. Erector spinae plane block (ESPB) is a simple, novel ultrasound-guided block technique, and its use has increased because the procedure is convenient and relatively safe. Although several cases have reported the zoster-associated pain (ZAP) control effect of ESPB, the efficacy of ESPB has not been compared with that of other types of nerve blocks for managing ZAP. This study aimed to compare the efficacy of ESPB with that of other types of nerve blocks for managing ZAP. *Study Design:* Retrospective case–control study. *Materials and Methods:* Medical records of 53 patients with acute thoracic herpes zoster were reviewed. We divided the participants into two groups: patients who received transforaminal epidural injection (TFEI) (*n* = 32) and those who received ESPB (*n* = 21). The efficacy of the procedure was assessed by a numerical rating scale (NRS) and by recording patient medication doses before the procedure and at 1 week, 1 month, 2 months, and 3 months after the procedure. *Results:* The time required for pain intensity to decrease to NRS ≤ 2 was not significantly different between the groups. The rate of medication discontinuation also was not different between the groups. There was no significant difference between the two groups in the proportion of clinically significant PHN (NRS ≥ 3) at any time point. *Limitations:* The relatively small sample size from a single center and the retrospective nature of the study served as limitations. *Conclusions:* The clinical effects of ESPB and TFEI were similar in patients with acute thoracic herpes zoster. ESPB could be considered an interventional option for ZAP management.

## 1. Introduction

The lifetime incidence of herpes zoster is about 30% [1]. Nerve damage and inflammation caused by varicella zoster virus (VZV) induce pain and could interfere with daily activity and quality of life [2,3,4]. If adequate pain reduction is not achieved in the acute phase of herpes zoster, sustained nociceptive input can be transmitted to the spinal cord. It may lead to neuropathic pain processing, such as central sensitization, which is thought to be one of the major causes of postherpetic neuralgia (PHN) [5,6]. In general, the acute stage of herpes zoster is defined as within 1 month after the rash occurs. Although there is no clear consensus on when to consider PHN after the development of herpes zoster, pain that persists for more than 90 days after the onset of acute zoster rash is generally considered PHN. Ref. [7] defines subacute herpes zoster as the presence of zoster symptoms beyond the acute phase and before PHN is diagnosed [8]. 

Appropriate antiviral medications along with oral analgesics can be used to control herpes zoster-associated pain (ZAP) in the acute phase. However, there are many cases where oral medication alone does not provide sufficient pain reduction.

In an actual clinical setting, various nerve blocks can be tried to control ZAP to prevent progression to PHN during the acute phase of herpes zoster. Studies have reported that sustained nociceptive input can be reduced in the acute phase via various blocks such as an epidural block or paravertebral block [9,10,11,12].

The dorsal root ganglion (DRG) is the location where the latent varicella zoster virus lies dormant and DRG is a sensory ganglion and contains many receptor channels. Therefore, it can be a primary target of nerve blocks for inhibiting ZAP [4]. 

Transforaminal epidural injection (TFEI) is a widely used epidural block method that has superior accessibility to the DRG, which is the essential location of pain signaling in herpes zoster [13]. It has been reported that applying TFEI in the acute phase of herpes zoster is a useful option for ZAP control and preventing chronic neuropathic pain such as PHN [13].

Erector spinae plane block (ESPB) is a novel ultrasound-guided block technique and it was first introduced in 2016 [14]. The analgesic effect of ESPB is achieved when local anesthetics (LA) enter the paravertebral space through the costotransverse foramen or intertransverse connective tissue complexes [15]. Because the ESPB procedure is convenient and relatively safe compared to neuraxial block techniques such as epidural block, its use for various pain conditions is increasing. ESPB is mainly used for thoracic postoperative pain management [16]. The efficacy of ESP block at lower thoracic levels for pain control after abdominal surgery also has been reported [17]. It has also been reported that a sympatholytic effect can be achieved through ESPB at a high thoracic level in neuropathic pain conditions such as complex regional pain syndrome (CRPS) [18].

In herpes zoster, one of the representative diseases that cause thoracic pain, several cases have been reported on the ZAP control effect of ESPB [19,20,21]. However, the efficacy of ESPB has not been compared with that of other types of nerve blocks for managing ZAP. In this study, to compare the effectiveness of TFEI and ESPB for managing ZAP, we analyzed the medical records of patients with acute thoracic herpes zoster who received TFEI or ESPB.

## 2. Methods

### 2.1. Participants

This study was approved by the Institutional Review Board of The Catholic University Daejeon St. Mary’s Hospital (DC21RISI0035). This trial was registered in the Clinical Trial Registry of Korea (trial registration number KCT0006485) before the initiation of the analysis. Medical records were collected from patients with acute herpes zoster who underwent TFEI or ESPB for control of ZAP from January 2017 to February 2021. As mentioned in the introduction, the acute phase is considered as the time from onset of skin lesions to 1 month after. Therefore, cases where the procedures were conducted more than 1 month after zoster onset were excluded from the analysis. 

At our institution, a nerve block is attempted when the numerical rating scale (NRS) is 3 or higher, and TFEI is usually used to treat ZAP. However, from 2017, ESPB has been applied as a ZAP treatment as well. This study assessed cases where either TFEI or ESPB were administered in patients over 19 years of age with moderate to severe ZAP. Patients with infection at the site of injection, coagulopathies, and pregnancy were not indicated for the procedure. 

Since ESPB generally is suitable for pain management of the thoracoabdominal region, ESPB mainly was performed for ZAP patients with thoracic dermatome. Therefore, to better analyze the effects of ESPB, this study only included patients with herpes zoster that affected the thoracic dermatome. Cases with zoster affecting the cervical level, lumbar level, and facial zoster were not included.

In many studies, clinically meaningful pain intensity is considered as the presence of pain with a numerical rating scale (NRS) ≥ 3 [22,23,24]. This study also used a pain intensity of NRS ≥ 3 as an indication of the procedure. If the worst pain in the last 24 h was NRS ≥ 3, it was recommended that the procedure be repeated. If pain intensity decreased to NRS ≤ 2, the procedure was not recommended, and self-tapering of the medication was recommended unless the pain increased again. 

Only medical records of patients with either ESP or TFEI applied were included in this study. Cases where more than two types of blocks were administered to one patient were not included. 

Medical records for up to 3 months after the procedure were analyzed. Cases where follow-up was missed or terminated before the pain decreased to NRS ≤ 2 were also not included.

All patients received appropriate antiviral treatment. Depending on the patient’s symptoms, the patient was treated with appropriate analgesics, anticonvulsants such as gabapentinoids, and tricyclic antidepressants.

### 2.2. Procedures

All procedures were performed by a single pain physician who was board-certified in anesthesiology pain medicine and had more than 7 years of experience in pain practice (E.D. Kim). All procedures were performed after receiving written informed consent from each patient.

#### 2.2.1. TFEI

For the TFEI procedure, the patient was placed in a prone position on a fluoroscopic-compatible table. The needle entry site was prepared and draped in a sterile manner. After local infiltration, a fluoroscope (ARCADIS Orbic, Siemens AG, Munich, Germany) was rotated obliquely toward the ipsilateral side. Then, a 22-gauge Tuohy needle (Neurotic Nerve Block Needle, Hakko, Nagano, Japan) was introduced inferior to the pars interarticularis under fluoroscopic guidance at the level of pathology. 

**Figure 1:**
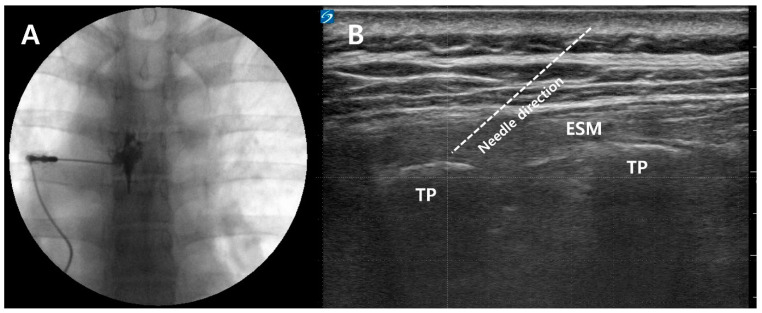
Fluoroscopic image of TFEI (**A**) and ultrasound images of ESPB (**B**). TP: Transverse process. ESM: Erector spinae muscle. TFEI: Transforaminal epidural injection. ESPB: Erector spinae plane block.

The needle tip position was adjusted so that it was inferior to the pedicle in the anteroposterior view (Figure 1A). In the lateral view of the fluoroscopic image, the needle tip was positioned in the lower posterior portion of the intervertebral foramen to avoid the possibility of vascular injury.

A total of 2–3 mL of contrast medium was used to confirm the position of the needle tip. Next, 5 mL of a mixture of 0.5% lidocaine and 5 mg dexamethasone was injected. 

#### 2.2.2. ESPB

The patients were placed in the prone position and the needle entry site was prepared and draped in a sterile manner. A high-frequency (12–15 MHz) linear probe (X-Porte, Sonosite, Bothell, MA, USA) was placed longitudinally on the affected side transverse process (TP) at the level of pathology. 

After confirming the TP, a 22-gauge Tuohy needle (Neurotic Nerve Block Needle, Hakko, Nagano, Japan) was inserted to contact the TP using the in-plane technique (Figure 1B). After bone-touching, hydrodissection with 1 mL of saline was used to confirm injectate spread between the TP and erector spinae muscle. Then, a total of 10 mL of a mixture of 0.5% lidocaine and 5 mg dexamethasone was injected. 

### 2.3. Data Collection

The following data were collected from medical records and analyzed: age, sex, involved level, days from zoster onset to the first procedure, NRS before the first procedure, NRS at 1 week and at 1–3 months after the procedure, number of procedures performed during the review period, and doses of anticonvulsants and analgesics before and at 1 week and at 1–3 months after the procedure. To facilitate the analysis, the doses of anticonvulsants and analgesics were converted to pregabalin-equivalent doses [25,26] and oral-morphine-equivalent doses [27], respectively.

### 2.4. Outcome Measures

We divided the participants into two groups: patients who received TFEI and those who received ESPB. We evaluated the NRS, the doses of analgesics and anticonvulsants at each time point, and the time required for pain intensity to decrease to NRS ≤ 2 to compare the clinical efficacy of the two blocks. The number of patients who were able to discontinue medication due to sufficient pain reduction was compared between the two groups. As in previous studies, we considered pain intensity NRS ≥ 3 as a clinically meaningful PHN and compared the proportion of clinically meaningful PHN values between the two groups [23,24].

### 2.5. Statistical Analysis

Data are presented as mean ± standard deviation (SD) for continuous variables. Data normality was evaluated using the Kolmogorov–Smirnov test. The distribution of time-to-event efficacy endpoints was accessed by the Kaplan–Meier product limit survival method, and differences between the two groups were compared using the log-rank test. The Mann–Whitney U test or the independent t-test was used to compare the outcomes between the two groups for continuous variables, whereas the Chi-square test or Fisher’s exact test was used for categorical variables.

## 3. Results

**Figure 2:**
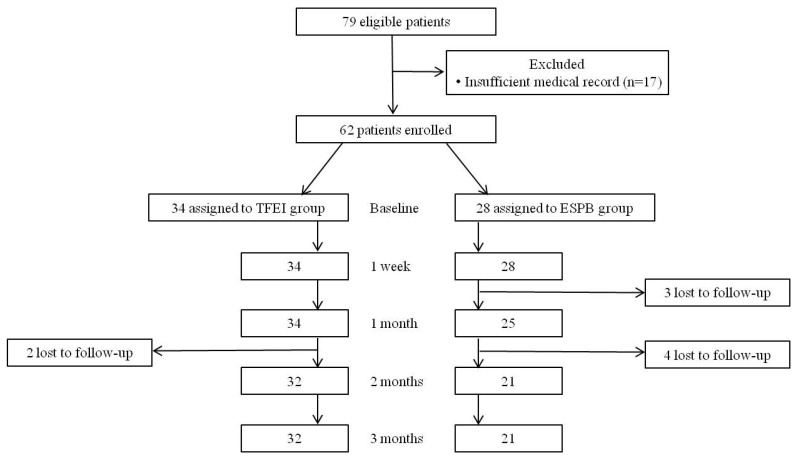
Flow diagram of the study subjects. TFEI: Transforaminal epidural injection. ESPB: Erector spinae plane block.

Medical records of 79 patients who met the inclusion criteria were reviewed, and the records of 17 patients were confirmed as insufficient so were excluded from the analysis. Nine patients were lost to follow-up before the NRS decreased to <3 within the review period. As a result, the medical records of 53 patients were available for a complete review for 3 months after the first block (TFEI or ESPB) (Figure 2). 

There were no significant differences in demographic data including age, sex, involved dermatome, and direction between the groups. There was also no significant difference between the groups in the underlying diseases of the study participants. NRS before the procedure was 7.06 ± 1.61 and 6.48 ± 1.54 for the TFEI group and ESPB group, respectively: this difference was not significant. Time from zoster onset to initiation of the procedure was 12.50 ± 8.55 days in the TFEI group and 17.25 ± 12.52 days in the ESPB group, which was not significantly different. The number of procedures performed during the review period was 2.00 ± 1.04 in the TFEI group and 2.36 ± 1.98 in the ESPB group, with no significant difference between the groups (Table 1).

**Figure 3:**
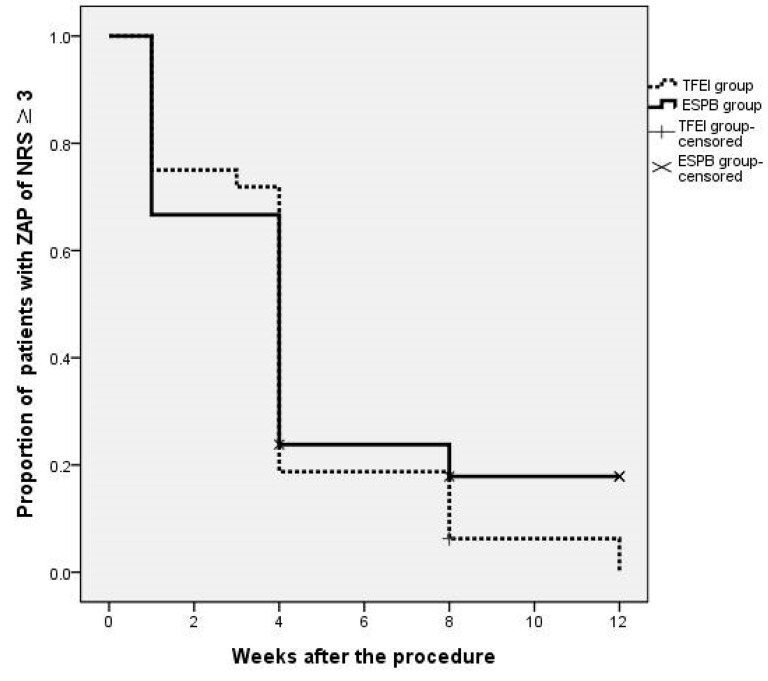
Time required for pain intensity to decrease to NRS ≤ 2. The number of censored patients was three at the 1-month and six at the 2-month (a total of 9 patients). NRS: Numeric rating scale. TFEI: Transforaminal epidural injection. ESPB: Erector spinae plane block. ZAP: Zoster-associated pain.

For patients who reported ZAP persistence, the NRS at 1 week and NRS from 1–3 months were not significantly different between the two groups (Table 2). The time required for pain intensity to decrease to NRS ≤ 2 was not significantly different between the groups (4.22 ± 0.52 weeks for the TFEI group and 4.67 ± 0.85 weeks for the ESPB group, *p* = 0.443) (Figure 3). 

**Figure 4:**
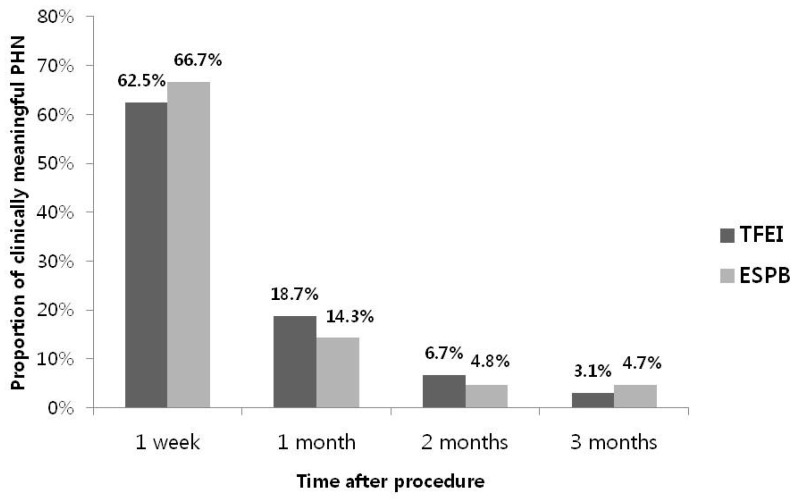
The proportion of patients with clinically meaningful PHN (NRS ≥ 3) over time. NRS: Numeric rating scale. TFEI: Transforaminal epidural injection. ESPB: Erector spinae plane block. PHN: Postherpetic neuralgia.

There was no significant difference between the two groups in the proportion of clinically significant PHN at all time points after the procedure (Figure 4). Pregabalin doses were significantly lower in the ESPB group than in the TFEI group at 1 and 2 months after the procedure. However, the analgesic doses did not differ between the two groups at any time point (Table 3). The rates of medication discontinuation were not significantly different between the two groups for either the anticonvulsants or analgesics (Table 4).

## 4. Discussion

In this study, no significant difference in NRS or proportion of PHN was noted between the two block methods at any time point after the procedure. The medication doses were compared only in patients who were still taking the drug and had persistent ZAP. The pregabalin-equivalent dose was significantly lower in the ESPB group at 1 month and 2 months after the procedure; however, the number of patients still taking medication was too small, and the results are difficult to regard as clinically meaningful. Instead, we hypothesize that a comparison of the proportion of patients who were able to stop taking medication is more clinically meaningful than a direct dose comparison of the drug. A comparison of the proportion of patients who were able to stop taking medication as an indicator for comparing clinical outcomes has been used in several previous studies [14,28]. The proportion of patients who were able to stop taking medication due to sufficient pain reduction was similar between the two groups. 

The time until the pain intensity decreased to less than 2 in NRS was not significantly different between the two block methods. In theory, because the transforaminal approach is more accessible to DRG where the VZV is being replicated and induces the ZAP [14], it was likely that TFEI would be associated with superior outcomes. However, the critical vessels are often located in the pathway of the needle during TFEI. Severe complications such as epidural hematoma or spinal cord infarction can occur due to vessel injury. 

Considering that the pathophysiology of herpes zoster is caused by decreased immunity, patients who require nerve block due to severe ZAP could have many underlying medical conditions [3,4,5,6]. Therefore, a neuraxial block such as TFEI might be difficult to implement in many cases.

In general, in acute herpes zoster occurring within 1 month after the onset of zoster rash, neuropathic changes such as central sensitization might not yet be established. If time passes without sufficient pain reduction after zoster onset, neuropathic changes starting in damaged sensory ganglion might become more severe [8]. Therefore, finding appropriate interventions during this acute period is very important to prevent the development of chronic neuropathic conditions such as PHN.

Paravertebral block has the characteristics of a dual block that can perform both somatic and sympathetic block, and the positive effects of paravertebral block for ZAP control and its effectiveness in preventing PHN have been reported [11,12,29]. Although anatomical studies have not reported consistent conclusions about whether injectate can enter the paravertebral space in ESPB [15], it is believed that during ESPB, the LA may enter the paravertebral space through the costotransverse foramen and the porous intertransverse connective tissue complex [30]. 

There are already several reports observing the effectiveness of ESPB for ZAP control [19,20,21]. The results of our analysis could be considered as indirect evidence for accessibility of the injectate to the paravertebral space which contains spinal root or DRG during ESPB implementation.

It is known that the sympathetic system can play an important role in the progression of neuropathic pain, and PHN is one of the representative neuropathic conditions. Although there is no clear evidence as to whether local anesthetics injected during ESPB can reach the sympathetic chain, the effectiveness of ESPB has been reported in neuropathic conditions such as CRPS and functional abdominal pain [18,31]. This suggests that ESPB also includes the characteristics of sympathetic block. Therefore, it is thought that the application of ESPB during the acute herpes zoster phase may contribute to preventing progression to PHN.

ESPB is technically very easy and is a relatively safe procedure that aims to inject therapeutic drugs into the interfascial plane between the TP and the erector spinae muscle after the block needle touches the TP of the target level of the vertebrae. 

Neuraxial blockade techniques, such as TFEI or interlaminar epidural block, may be accompanied by hemodynamic changes and therefore may not be appropriate for patients with a variety of medical problems. In these patients, ESPB could be considered as the interventional option for ZAP control, with relatively little burden or risk for complications.

Additionally, herpes zoster patients often take anticoagulants for various medical reasons. In order for these patients to undergo neuraxial block such as TFEI, the procedure must be performed after discontinuing the administration of anticoagulant for a certain period of time to prevent critical complications such as epidural hematoma. In these cases, the procedure might be performed relatively late, and the delay in the procedure could result in missing the golden time to prevent progression to PHN. Recently, an analysis was published showing that the risk of bleeding is extremely low or non-existent when performing ESPB in patients taking anticoagulants [32]. Therefore, in such clinical situations, ESPB might be tried first instead of neuraxial block methods. As a result, the progression to PHN may be reduced because the procedure can be performed earlier. Moreover, in the present analysis, the TFEI and ESPB procedures had similar clinical results.

Although paravertebral block might be considered relatively safer than neuraxial blocks such as TFEI, it could still be difficult to apply in vulnerable patient groups, therefore future research comparing the effectiveness of paravertebral block and ESPB in ZAP control is also considered necessary.

In general, if the effect of a single block is insufficient, a continuous block through catheterization could be considered. Epidural catheterization for ZAP control has been reported as an effective approach [33,34]; however, catheterization into the epidural space can be associated with potential risks, such as infection. Moreover, in patients with various underlying diseases, epidural catheterization can be even more dangerous. Just as ESPB is considered safer than neuraxial block methods such as epidural block, continuous ESPB via catheterization can also be considered relatively safer than continuous neuraxial block.

The effectiveness of continuous ESPB on functional abdominal pain and CRPS has already been reported [18,31]. Based on the results of the present analysis, additional research might be conducted to confirm the effectiveness of continuous ESPB in ZAP management.

This study had several limitations. Because this was a retrospective analysis, it was not possible to enforce a controlled environment for TFEI or ESPB implementation. Also, because the sample size was extracted retrospectively based on available data from a single medical center, statistical potency may have been insufficient. In the future, a comparative study between ESPB and other block methods through a non-inferiority test or superiority test with a statistically appropriate sample size is also necessary.

The standard injection volume of ESPB has not yet been determined, but according to previous studies, a volume of 10 to 20 mL is used [14,15,16,17,18,19,20,21]. We used a volume of 10 mL to deliver the injection into the paravertebral space while minimizing the spread of the injectate to other areas. In order to find the optimal ESPB regimen for managing ZAP, a comparative study according to the injection volume when performing ESPB might be conducted in the future.

In this study, only cases in which procedures were performed on thoracic lesions were included. However, some studies have reported positive effects by applying ESPB to lumbar lesions [35,36]; therefore, in the future, studies comparing ESPB and TFEI may be conducted in patients with herpes zoster occurring at other levels such as lumbar level.

## 5. Conclusions

In conclusion, the clinical effects of ESPB and TFEI were similar in patients with acute thoracic herpes zoster. ESPB could be considered an interventional option for ZAP management. To verify the results of this study, further prospective studies in patients with ZAP are needed to determine which type of block more facilitated pain reduction. 

## Figures and Tables

**Table 1:** Demographic data of participants.

	TFEI Group (*n* = 32)	ESPB Group (*n* = 21)	*p*-Value
Age ± SD, years	59.79 ± 14.28	66.46 ± 12.57	0.058
Gender (male/female)	12/20	6/15	0.502
Direction (right/left)	17/15	8/13	0.284
NRS before procedure	7.06 ± 1.61 (95% CI: 6.47–7.59)	6.48 ± 1.54 (95% CI: 5.71–6.94)	0.586
Dermatome			
Chest area (T1–4)	16	14	0.142
Below chest to umbilicus (T5–10)	11	7	
Below umbilicus (T11,12)	5	0	
Underlying disease			
cardiovascular	7	6	0.739
Diabetes	3	3	
Thyroid	2	0	
Combined	4	3	
None	16	9	
Time to the first procedure from zoster onset, days	12.50 ± 8.55	17.25 ± 12.52	0.082
Number of procedures during the review period	2.00 ± 1.04	2.36 ± 1.98	0.367

SD: Standard deviation. CI: Confidence interval. NRS: Numeric rating scale. TFEI: Transforaminal epidural injection. ESPB: Erector spinae plane block.

**Table 2:** Pain intensity (NRS) of patients with remaining ZAP at each time point.

Time Point	TFEI Group	ESPB Group	*p*-Value
1 week	3.13 ± 1.81, *n* = 34 (95% CI: 2.53–3.76)	2.95 ± 2.01, *n* = 28 (95% CI: 2.55–4.10)	0.747
1 month	2.00 ± 1.63, *n* = 29 (95% CI: 1.40–2.60)	2.05 ± 2.01, *n* = 18 (95% CI: 1.14–2.95)	0.201
2 months	1.40 ± 1.71, *n* = 10 (95% CI: 0.17–2.63)	2.00 ± 0.82, *n* = 4 (95% CI: 0.70–3.30)	0.522
3 months	3.00 ± 1.73, *n* = 3 (95% CI: −1.30–7.30)	3.00, *n* = 1	1.000

NRS: Numeric rating scale. TFEI: Transforaminal epidural injection. ESPB: Erector spinae plane block. ZAP: Zoster-associated pain. CI: Confidence interval.

**Table 3:** Doses of medication at each time point.

	Anticonvulsant (Pregabalin-Equivalent Dose)			Analgesics (Morphine-Equivalent Dose)		
	TFEI Group	ESPB Group	*p*-Value	TFEI Group	ESPB Group	*p*-Value
Pre-procedure	54.06 ± 42.99, *n* = 32	57.62 ± 46.98, *n* = 21	0.778	19.79± 5.27, *n* = 31	21.00 ± 3.16, *n* = 21	0.516
1 week	120.00 ± 71.58, *n* = 31	114.29 ± 95.22, *n* = 21	0.806	19.57 ± 9.08, *n* = 31	21.92 ± 2.08, *n* = 21	0.202
1 month	186.90 ± 104.14, *n* = 29	127.50 ± 83.61, *n* = 15	0.048 *	18.26 ± 9.74, *n* = 26	14.14 ± 10.40, *n* = 13	0.223
2 months	235.71 ± 75.00, *n* = 7	75.00 ± 0.00, *n* = 2	0.031 *	23.50 ± 13.09, *n* = 6	22.50, *n* = 1	0.644
3 months	250.00 ± 86. 60, *n* = 3	75.00, *n* = 1	0.222	23.75 ± 18.49, *n* = 3	0.00, *n* = 0	0.382

TFEI: Transforaminal epidural injection. ESPB: Erector spinae plane block. *: *p* < 0.05.

**Table 4:** The proportion of patients who were able to discontinue the medication.

	Anticonvulsant			Analgesics		
	TFEI Group, *n* (%)	ESPB Group, *n* (%)	*p*-Value	TFEI Group, *n* (%)	ESPB Group, *n* (%)	*p*-Value
1 week	1/31 (3.12%)	0/21 (0.00%)	0.413	1/31 (3.12%)	0/21 (0.00%)	0.413
1 month	3/29 (9.37%)	6/15 (28.57%)	0.069	6/26 (18.75%)	8/13 (38.09%)	0.202
2 months	25/7 (78.13%)	19/2 (90.48%)	0.126	26/6 (81.25%)	20/1 (95.24%)	0.223
3 months	29/3 (90.63%)	20/1 (95.24%)	0.644	29/3 (90.63%)	21/0 (100.00%)	0.149

TFEI: Transforaminal epidural injection. ESPB: Erector spinae plane block.

## Data Availability

The data that supports the finding of this study are available from the corresponding author upon reasonable request.
